# Post-Surgical Inflammatory Neuropathy: An Underappreciated but Critical and Treatable Cause of Postoperative Neuropathy

**DOI:** 10.7759/cureus.11927

**Published:** 2020-12-05

**Authors:** Christopher A Godlewski, Hari Kalagara, Rocio Vazquez Do Campo, Theresa Northern, Promil Kukreja

**Affiliations:** 1 Anesthesiology and Perioperative Medicine, University of Alabama at Birmingham, Birmingham, USA; 2 Neurology, University of Alabama at Birmingham School of Medicine, Birmingham, USA

**Keywords:** inflammatory neuropathy, total hip arthroplasty, quadratus lumborum block, interfascial plane block, nerve conduction study, peri-operative, nerve injury

## Abstract

The diagnosis and management of postoperative nerve injury can be a challenging and frustrating proposition for the patient, surgeon, and anesthesia provider. Unfortunately, in many cases, the true etiology is never elucidated and the injury is ascribed to positioning or a nerve block with “expectant management” being the order of the day, which can result in persistent disability for the patient. However, there is a rare subset of disorders affecting the nervous system that can masquerade as a peripheral nerve injury that warrants further investigation of risk factors and co-morbidities when other common causes of nerve injury are ruled out. We describe a patient with rheumatoid arthritis that underwent revision hip arthroplasty and presented almost immediately in the postoperative period with what was initially diagnosed as femoral nerve palsy. Further diagnostic workup later revealed that she had suffered from postoperative inflammatory neuropathy resulting in lumbosacral plexus injury and not a discrete nerve injury. Had the true cause been identified early enough, treatment with corticosteroids could have been initiated in an attempt to mitigate and perhaps reverse the progress of the neuropathy. We present this cautionary tale to remind practitioners to continue to be vigilant and consider more esoteric and unconventional diagnoses in the workup of perioperative neuropathies.

## Introduction

Total hip arthroplasty (THA) is now the second-most common joint replacement surgery in the US due in part to an aging population [1]. Opioid-sparing analgesic treatments, such as lumbar plexus and femoral nerve blocks, are effective, but they carry a high risk of undesirable lower limb motor or muscle weakness. The quadratus lumborum (QL) block is a relatively new truncal interfascial plane block regional technique that provides effective pain control after upper and lower abdominal surgeries, with efficacy also being shown for total hip arthroplasty [2]. The quadratus lumborum muscle is encased between the middle and anterior thoracolumbar fascia, which communicates with the fascia of the psoas major muscle medially and with the transversalis fascia laterally [3]. Local anesthetic injected between the quadratus lumborum muscle and a layer of the thoracolumbar fascia can potentially spread to the thoracic paravertebral space [4] and may spread to indirectly block the lumbar plexus.

Nerve injuries are a rare complication after THA and may result in severe adverse outcomes. The reported incidence of nerve injuries after THA ranges from 0.6-3.7%, with a much higher incidence (7.6%) among revision THA patients [5-6]. Femoral nerve palsy is the second most common nerve injury associated with THA. The cause of nerve injury can be attributed to multiple factors, including causes from surgery and anesthesia but also at times from primarily patient-related factors. Besides patient discomfort and dissatisfaction, these injuries may result in significant medicolegal consequences for the physician care team. It can often be quite difficult to pinpoint an exact etiology for postoperative neuropathy or discrete nerve injury; very often, by default, the injury is ascribed either to the regional anesthesia or surgical technique or patient positioning, with a conclusive cause usually being unknown or unclear. However, though rare, a certain subset of these injuries can be idiopathic or related to patient comorbidities; importantly, some may be treated to halt or reverse progression if these more uncommon diagnoses are considered at an early stage. This warrants a careful and thorough assessment of risk factors and possible causative etiologies to implement preventive measures in a timely manner.

 As a cautionary tale, we present the following case report of primarily a postoperative femoral nerve palsy with other subtle findings to encourage education and consideration of these “red herring” diagnoses to facilitate recognition and timely diagnosis and treatment.

## Case presentation

The patient is a 50-year-old Caucasian female with a past medical history of HLA B27-positive juvenile rheumatoid arthritis (JRA) presenting for the second revision of her left total hip arthroplasty secondary to pain and polyethylene wear. As a result of her JRA treatment, she had steroid-induced osteonecrosis of both hips, which had required bilateral hip replacements many years prior to presentation. She had undergone left total hip revision some 20 years prior at another institution. She was a former smoker and weighed 68 kg with a body mass index (BMI) of 25. Preoperatively, she denied any numbness, weakness, or any other neurologic findings in any of her extremities. She underwent preoperative placement of a left posterior quadratus lumborum block (QLB) using 30 cc of 0.5% ropivacaine with 2 mg of dexamethasone added with general endotracheal anesthesia for her surgery. As a result of her body habitus, ultrasound imaging proceeded without ambiguity, and the local anesthetic was injected into the fascial plane along the posterior border of the quadratus lumborum. On her first postoperative day, the patient complained of pain and dysesthesias in the left knee; she had 0/5 strength in left hip flexion and knee extension with substantially decreased sensation to her distal anterior/anteromedial thigh and knee. Initial evaluation by physical therapy documented 0/5 quadriceps strength. For her protection, she was placed in a knee immobilizer. She continued to have numbness over her anterior knee. At her first postoperative visit on day 15, the operative extremity that previously had been shorter than the contralateral was found to now be ¾” longer than the contralateral, and she was given an orthotic to level her pelvis.

She underwent nerve conduction studies as well as electromyography seven weeks after surgery; the physical exam of the operative extremity performed by the neurologist at that time demonstrated 2/5 strength in her hip flexors, 1/5 knee extension, 4+/5 ankle dorsiflexion, hip abduction, and knee flexion. Hip adduction and ankle plantarflexion were normal. Her sensation was reduced in the L2-L4 dermatomes with an absent patellar reflex and a mildly reduced Achilles’ reflex. Of note, she was also found to have an area of hyperesthesia/contact allodynia over the anterolateral thigh. Nerve conduction studies of the left lower limb were normal except for mildly prolonged peroneal and tibial F waves. Needle electromyography (EMG) demonstrated profuse denervation and no voluntary motor activation of vastus medialis and iliopsoas muscles. The adductor longus, tensor fasciae latae, gluteus maximus, and L4 paraspinal muscles demonstrated mild changes of subacute reinnervation. The electrodiagnostic findings were consistent with a subacute left lumbosacral radiculoplexus neuropathy with more severe involvement of the femoral nerve. The patchy involvement of the lumbosacral plexus was not well explained by a nerve block, surgical trauma, or patient positioning intraoperatively, and it is often seen in inflammatory neuropathies.

## Discussion

To the best of our knowledge, this is the first case report to describe this unexpected complication of perioperative neuropathy after posterior QL block performed for revision THA analgesia. Perioperative nerve injuries are rare, yet devastating complications that present difficult problems challenging both anesthesiologists and surgeons. New weakness with pain and numbness after surgery is mainly attributed to a nerve block by default before considering other causes. The common causes of perioperative nerve injury are surgical trauma, intraoperative positioning, or regional anesthetic techniques. The uncommon causes of postsurgical neuropathy are rarely considered by anesthesia and surgery providers. In the absence of any obvious surgical or anesthesia factors and based on the EMG studies, the diagnosis was suggestive of neuropathy. Postsurgical inflammatory neuropathy is an underappreciated cause of postoperative nerve injury [7].

The QL block is a relatively new block now commonly used for lower abdominal surgeries like cesarean section. The main advantages of the QL block mentioned in recent literature are effective pain control, opioid-sparing analgesic effect, and preservation of lower limb muscle strength, which in the era of accelerated physical therapy after THA are encouraging [2]. Multiple regional techniques have been used in the past, but there is not a “best-proven intervention” for THA analgesia [8]. The main regional techniques for THA include lumbar plexus block, lumbar epidural, femoral nerve block, sciatic nerve block, fascia iliaca block, pericapsular injection, or obturator nerve block. Unfortunately, all the above-mentioned blocks provide either inconsistent or partial analgesia or are associated with lower extremity weakness that may interfere with physical therapy or increase the risk of falls. The use of a lumbar epidural catheter or inadvertent epidural spread of a lumbar plexus block can result in hypotension, leg weakness, and related adverse effects. The peripheral nerve blocks have been shown to be associated with falls after knee and hip arthroplasty [9]. The anterior approach for the QL block has also been shown to result in lower extremity weakness [10].

The lumbar paravertebral block at L1 and L2 for postoperative analgesia for hip arthroscopy has demonstrated motor weakness in hip flexion and knee extension using a small dose of 5 ml of 0.5% ropivacaine at each level [11]. In this case report, we used a much higher dose of 30 ml of 0.5% ropivacaine. The optimum dose for the QL block is not validated, although some authors have seen equivalent analgesia with lower concentrations [12]. Higher concentrations are more likely to cause dense block with potential motor weakness, although low concentration blocks are also reported to cause prolonged motor weakness lasting greater than 48 hours [13]. The prolonged weakness without improvement is inconsistent with the effect of a nerve block.

The term anterior or transmuscular QL block refers to the injection of a local anesthetic in the tissue plane between the quadratus lumborum and psoas major [14]. The psoas major muscle, while housing the lumbosacral plexus, maybe a potential path of local anesthetic spread from the QL block to the lumbar plexus. The femoral nerve and obturator nerve from the lumbar plexus innervate the anterolateral and anteromedial hip capsule, respectively [15]. The anterior QL block aims to indirectly block the lumbar plexus branches and potentially causes quadriceps weakness. In this case, a posterior QL block approach was used, where the local anesthetic was injected farther away from the lumbar plexus and hence may not be responsible for prolonged leg weakness.

The patient in this case report presented with an acute onset of significant quadriceps weakness, neuropathic pain, and sensory loss in the femoral nerve distribution in the immediate postoperative period after hip surgery. There was no improvement in weakness within the first six to eight weeks and mechanical factors and complications from anesthesia were reasonably excluded. Laughlin et al. reported on seven patients, with a presentation similar to our patient, who developed ipsilateral leg weakness within a month of hip surgery due to inflammation of lumbosacral plexus and findings on nerve biopsy consistent with microvasculitis [16]. Previously, the same group reported their tertiary center experience with 33 patients diagnosed with postsurgical inflammatory neuropathies based on clinical features and nerve biopsy, EMG, and magnetic resonance imaging (MRI) findings.

Postsurgical inflammatory neuropathies are likely under-recognized and should be considered in the presence of 1) spatio-temporal separation from the site and time of surgery (although some cases may present in the immediate postoperative period), 2) progression of weakness or pain postoperatively and no clinical improvement within the first postoperative month, 3) severe neuropathic pain, and 4) patchy involvement of muscles on EMG.

Risk factors for postsurgical inflammatory neuropathy may include diabetes mellitus, genetic predisposition, and pre-existing inflammation or neuropathy. The surgical process, volatile anesthetic use, or recent blood transfusion may also be contributing [17]. Prompt recognition and administration of intravenous corticosteroids in the acute phase can lessen the duration and severity of the symptoms [18].

Femoral nerve injury is a rare but devastating complication of the THA. The common mechanisms of femoral nerve injury during THA include prolonged hyperextension, hematoma compression in patients on anticoagulants, improper use of acetabular retractors, inadequate positioning, and cushioning during surgery [19]. The anterior THA approach is associated with a higher risk of nerve injury. The major risk factors, in this case, were revision THA, female gender, limb lengthening, and cement-less surgical technique [20]. The acute progression of leg weakness and prolonged course without improvement prompted for the neurology consultation. The EMG and nerve conduction studies along with clinical presentation were suggestive of inflammatory neve insult.

The number of case reports or published studies to date are insufficient to discern the actual incidence of this particular complication. The delayed recovery and added hospital costs of unplanned admission warrants further investigation to elucidate the factors responsible. Ideally, a large prospective study would be helpful to identify inflammatory neuropathy risk factors like patient co-morbidities, surgical positioning, surgical technique, anesthesia type, and surgery duration.

## Conclusions

In this case report, we demonstrate that inflammatory mechanisms of nerve injury should be investigated early when other causes like mechanical insult are ambiguous. Early identification of these patients through clinical neurology evaluation, EMG study, and nerve biopsy, and the prompt initiation of treatment with corticosteroids may lead to improved outcomes.
